# Improving emotion recognition is associated with subsequent mental health and well-being in children with severe behavioural problems

**DOI:** 10.1007/s00787-020-01652-y

**Published:** 2020-09-30

**Authors:** Amy E. Wells, Laura M. Hunnikin, Daniel P. Ash, Stephanie H. M. van Goozen

**Affiliations:** 1 School of Psychology, Cardiff University, Cardiff, UK; 2 Department of Clinical Child and Adolescent Studies, Leiden University, Leiden, The Netherlands; 3 Department of Criminology and Criminal Justice, University of Northampton, Northampton, UK

**Keywords:** Emotions, Facial expressions, Antisocial behaviour, Wellbeing, Intervention

## Abstract

Impaired emotion recognition is a transdiagnostic risk factor for a range of psychiatric disorders. It has been argued that improving emotion recognition may lead to improvements in behaviour and mental health, but supportive evidence is limited. We assessed emotion recognition and mental health following a brief and targeted computerised emotion recognition training in children referred into an intervention program because of severe family adversity and behavioural problems (*n* = 62; aged 7–10). While all children continued to receive their usual interventions, only children impaired in emotion recognition (*n* = 40) received the emotion training. Teachers blind to whether or not children had received the training rated children’s mental health problems before and 6 months after the training. Participants who received the emotion training significantly improved their recognition of negative and neutral facial expressions. Although both groups showed improved behaviour at follow-up, the reduction in behavioural problems was only significant in children who received the emotion training. Post-training emotion recognition scores predicted mental health problems 6 months later independently of initial emotion recognition ability and severity of behavioural problems. The results are consistent with the view that targeting emotion recognition can improve longer term functioning in individuals with disruptive behaviour, although further research using fully randomised designs is needed before causal conclusions can be drawn with confidence.

**Electronic supplementary material:**

The online version of this article (10.1007/s00787-020-01652-y) contains supplementary material, which is available to authorized users.

## Introduction

The ability to accurately recognise the emotions of others is crucial for interpersonal interactions and social functioning [18]. Emotion recognition is positively associated with the initiation and maintenance of healthy social relationships [27] and young children who are good at recognizing the emotions of others are more socially skilled and popular with their peers [11].

The degree to which emotion skills develop in childhood has significant implications for children’s later social competence. Difficulties with emotion recognition are well documented in children and adolescents exhibiting a range of mental health, neurodevelopmental and behavioural difficulties. In a systematic review, Collin et al. [6] found emotion recognition deficits in a wide range of child psychiatric disorders, including conduct disorder (CD), anxiety and attention deficit hyperactivity disorder (ADHD), indicating that impaired emotion recognition is a transdiagnostic risk factor. Interventions that successfully target and improve emotion recognition could therefore improve behaviour and social functioning and reduce the likelihood of a range of psychopathology, relieving pressure on statutory services [20].

Although an impairment in emotion recognition is a risk factor for many neurodevelopmental disorders, the impairments vary in nature. In antisocial behaviour (ASB), the impairment is specific to the recognition of negative emotions, especially fear [30], but impairments in sadness and anger have also been reported [12]. In addition, antisocial individuals appear to display a hostile attribution bias (HAB), misinterpreting ambiguous or neutral faces as angry [7]. While the majority of research finds antisocial individuals to show an impairment in emotion recognition, it is worth noting that there is considerable individual variation in emotion recognition ability (Eugene et al. 2003) and some research has found emotion recognition to be intact in antisocial individuals (Glass and Newman 2006). Therefore, not all individuals with behavioural problems are likely to be impaired in emotion recognition.

A few studies have attempted to correct emotion recognition impairments and thereby improve subsequent behaviour. Penton-Voak et al. [33] demonstrated that modifying hostility biases in young offenders was associated with fewer self- and staff-reported aggressive incidents up to 2 weeks after training. Similarly, Rawdon et al. [35] found that increasing the perception of happiness over disgust in ambiguous expressions in socially anxious adolescents led to fewer self-reported depressive symptoms 2 weeks later. However, these improvements in mood and behaviour were only short-term. The only study to date in which longer term effects of emotion training on objectively recorded behaviour were examined found that improving negative emotion recognition in juvenile offenders led to a significant reduction in severity of crimes committed up to 6 months later [20].

These previous studies were conducted in adolescents. To our knowledge, no studies have been conducted to date in children who have experienced severe adversity and display disruptive behaviour and as part of an early prevention; early intervention is the most (cost-) effective way to prevent and reduce mental health problems [40]. Emotion recognition skills continue to develop through the primary school years [36], providing an ideal time to intervene. Dadds et al. [7] found that emotion recognition training improved conduct problems in 6–16 years with callous-unemotional (CU) traits 6 months after receiving the training, despite there being no improvement in emotion recognition. Because parents were involved in both the training and reporting on behaviour, no independent assessments were available to verify these improvements.

The Research Domain Criteria (RDoC) approach [26] advocates the study of underlying and transdiagnostic processes involved in mental health problems and the development of personalised interventions targeting those with clear problems, rather than assuming that impaired processes are present in all those sharing a diagnosis or exhibiting similar behavioural problems. In line with this approach, it has been established that emotion recognition can be rapidly improved in children with behavioural problems [23]. Building on this work, in the current study we aimed to examine the longer-term effects of an emotion recognition training on behaviour and mental health 6 months later, as rated by teachers who were unaware which children had received emotion training.

The behaviour and well-being of children with behavioural problems were assessed at pre-test and at 6-month follow-up through independent teacher reports, using the Strengths and Difficulties Questionnaire (SDQ [17]). The SDQ is frequently used in epidemiological [31], longitudinal [39] and intervention [25] research. All children in the present study were included in an intervention programme and therefore received various forms of support. In line with RDoC guidelines [26], only those children with significant emotion recognition impairments received the emotion training, in addition to the other interventions.

## Method

All elements of the research were approved by the relevant institutional Research Ethics Committee. Informed written consent was provided by the participant’s parents/guardians and informed written assent was obtained from the participants.

### Participants

This study was part of a larger intervention project that aimed to identify socio-emotional impairments in children displaying behavioural problems (BP). Sixty-two children (52 male) aged 7–10 (*M* = 8.61, SD = 1.06) completed the pre- and post-test emotion recognition task and were followed up 6 months later.

All participants were part of an early intervention programme and had been referred into the study by teachers or police community support officers (PCSOs). The programme works with children from families known to the police or social services and who have been exposed to multiple adverse childhood experiences (ACEs). These experiences are linked to increased risk of negative outcomes in later life [34], including violence, mental illness and substance abuse [21]. Children in the programme do not have a formal mental health diagnosis but show behavioural and emotional problems and are close to the threshold for clinical help.

All children had received an initial comprehensive assessment completed by a police community support officer (PCSO) or police constable with the children’s parents/guardians. Following this assessment, each child had an individualised intervention plan devised for them, tailored to the child’s and their family’s circumstances. The interventions offered ranged from providing social, health, financial assistance and support for the family, to access to a range of interventions provided by the school and/or local authority, which were specifically designed to improve the well-being of the child. Thus, all children received interventions tailored to their specific circumstances, and those interventions were unique to each child: no child received the same set of interventions. As part of this individualised and tailored intervention plan, children who exhibited emotion recognition impairments received alongside the usual interventions our brief computerised training.

After referral, the child’s teacher completed the SDQ to confirm mental health status of the child over the last 6 months. Children scoring in the ‘slightly raised’ or ‘above’ range for key subscales were deemed to show behavioural problems and were eligible to take part. All participants then completed a facial emotion recognition (FER [24] test). Based on their FER performance, participants were assigned to either the behavioural problems with emotion training (BP+) group (*n* = 40) or the behavioural problems without emotion training (BP−) group (*n* = 22).

Participants in the BP+ group were found to have impaired emotion recognition, and would therefore be expected to benefit from the emotion training, because they scored equal to or less than 66.67% for at least one of fear, sadness, anger or neutral recognition, and these emotions play an important role in ASB (Bowen et al. 2014) [22]. This threshold is 1.5 standard deviations below the average score observed in a typically developing sample [24]. Consistent with the RDoC recommendation that interventions should be tailored to those individuals displaying an impairment [26], only participants in the BP+ group received the emotion training. However, all participants continued to receive their usual interventions delivered by the early intervention programme and the behaviour of all participants (BP+ and BP−) was followed up 6 months later.

Participant sample size was based on an a priori power calculation (G*Power 3.1; Faul et al. 2007) for a matched pairs *t* test with 90% power (*α* = 0.05) and effect size *d* = 0.58. The effect size was based on our previous work [20] that investigated the effect of emotion recognition training on criminal behaviour in young offenders.

## Materials

### Demographics and IQ

The Wechsler Abbreviated Scale of Intelligence, first edition (WASI [42]) was used to estimate IQ. Socioeconomic status (SES) was estimated using the Office of National Statistics estimates of average household total weekly income, based on residential postcode (Low = £0–£520; Middle = £571–£670; High = £671+ [20]).

### Strengths and difficulties questionnaire (SDQ)

The SDQ is a 25-item questionnaire comprising five subscales measuring behavioural, emotional and social difficulties and strengths [17]. Summing the subscales yields a ‘total difficulties’ score ranging from 0–40. This reflects children’s mental health and well-being and is used as *the* measure of mental health and well-being in reports by the Office for National Statistics (ONS [3]). The SDQ has been used to monitor the effectiveness of interventions and as a measure of health and well-being in community settings such as schools [15]. It has been shown to be a useful outcome measure in numerous samples, including in children at risk for developing conduct problems [25]. Goodman and Goodman [15] showed that the total difficulties score can be used as a dimensional measure of child mental health when comparing scores over time or after an intervention. The total difficulties score can also be analysed categorically. A total score of 12 or above is considered to be above average, and only 10% of the UK population is so classified [16].

Teacher-reported SDQ was used, because teachers are familiar with a range of children and have experience of normative child development. Perhaps as a result, teacher ratings have greater internal consistency and stability than parent ratings [14]. Importantly, in the current study, teachers were unaware of whether participants did or did not receive the emotion training. Cronbach’s *α* for the SDQ measure was 0.71 at pre-test and 0.84 at 6-month post-test, indicating good internal consistency.

### Facial emotion recognition (FER)

**Fig. 1:**
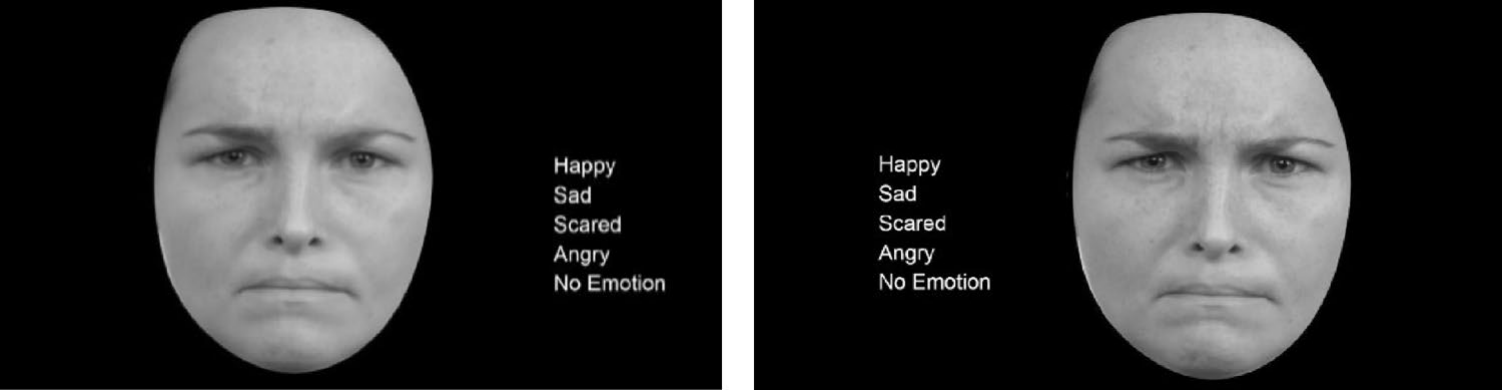
Example of a low (left) and high (right) intensity angry face from the FER task

The FER test was administered twice by a research assistant to measure children’s ability to recognise faces displaying happy, sad, fearful, angry and neutral expressions [24]. Children viewed 60 faces on a laptop computer, each displaying one of five expressions at low or high intensity. Each face was presented for three seconds, after which the question “What emotion (if any) is this person showing?” appeared, along with five emotion labels. Participants were asked to select a response (see Fig. 1 for example stimuli). Emotion recognition scores for each emotion were examined, as well as a composite negative emotion recognition score (calculated by taking the mean recognition score for fear, sadness and anger). A composite negative emotion score was used because of the role of negative emotion recognition impairments in the development of ASB; however, we had no specific expectations or hypotheses for the recognition of individual negative emotions.

### Cardiff emotion recognition training (CERT)

The CERT is a computerised emotion recognition training to improve the identification of facial expressions of happiness, sadness, fear and anger by directing attention to key facial features and providing assistance with the interpretation of these features [22] (https://emotionrecognition.cardiff.ac.uk/index.php). In addition to improving emotion recognition ability, the CERT also aims to improve the ability to understand when and why certain emotions are shown and that people can show different emotions in the same situation. It also provides guidance on the appropriate way to respond to someone displaying an emotion. The CERT consists of three 20-min sessions, delivered once a week over three consecutive weeks. It was delivered on a one-to-one basis by a family support worker or PCSO in a quiet room at the child’s school in addition to their usual interventions.

## Procedure

All elements of the study were completed at school. Participants completed the emotion recognition test twice: at pre-test (baseline) and again 8 weeks later (post-test). For those who received the CERT, the post-test emotion recognition test was completed 2 weeks after the third and final training session; for those who did not receive the training, the second session was 8 weeks after the first. Both groups continued to receive their standard interventions delivered by family support workers or PCSOs. Follow-up teacher SDQ ratings for all children were collected 6 months later.

### Statistical analyses

Possible demographic differences between the two groups were analysed using independent samples t-tests for continuous variables and *χ*^2^ tests for binary variables. Spearman’s correlations were used to determine the relationship between emotion recognition and behaviour at pre-test.

Percent correct for negative emotion recognition was calculated by taking the mean recognition score for sadness, fear and anger. Given the significant differences between the BP+ and BP− groups for FER and total SDQ score, separate paired samples *t *tests within each group were used to determine whether their emotion recognition ability differed between pre-test and post-test, and whether SDQ score differed between pre-test and 6-month follow-up. Where there was a significant difference in SDQ score between pre-test and 6-month follow-up, McNemar’s tests were used to determine whether there was a change in the percentage of participants with an above average number of total difficulties.

To assess whether change in emotion recognition was associated with change in behaviour, change scores were calculated for SDQ (pre-test minus 6-month follow-up) and FER (post-test score minus pre-test). For both the SDQ and FER, a positive score reflects improvement and a negative score reflects a decline. Where there was a significant difference in total SDQ score between pre-test and 6-month follow-up, Pearson’s correlations were calculated to assess the relation between FER change scores and SDQ change scores. Hierarchical regression was used to determine whether emotion recognition at post-test predicted behaviour at the 6-month follow-up independently of pre-test FER, pre-test SDQ and IQ.

Effect sizes for *t* tests are reported as Cohen’s *d*, those for ANOVAs are reported as partial eta squared ($\eta_{{\text{p}}}^{2}$), and those for multiple regression are reported as Cohen’s *f*^2^.

## Results

### Demographics

The BP+ and BP− groups did not differ in age, IQ, gender, or socioeconomic status. However, in addition to having a significantly lower FER score, the BP+ group had a significantly higher SDQ score at pre-test (see Table 1).

**Table 1:** Demographics, emotion recognition ability and behavioural characteristics

	BP+ (*n* = 40)	BP− (*n* = 22)	*p*
Age (years)	8.50 (1.11)	8.82 (.96)	0.262
IQ	82.58 (30.59)	95.36 (13.50)	0.07
Gender			0.264
% male	80	90.9	
% female	20	9.1	
SES			0.695
% low	8.3	13.6	
% medium	55.6	59.1	
% high	36.1	27.3	
Total FER (pre-test)	76.54 (12.43)	90.61 (4.26)	<0.001
Negative FER (pre-test)	71.22 (16.02)	89.13 (8.82)	<0.001
Total SDQ (pre-test)	18.79 (5.16)	15.04 (6.03)	0.013

Mean, with standard deviations in parentheses, or percentages are shown.

*IQ* intelligence quotient (two-subtest WASI), *SES* socioeconomic status, *FER* facial emotion recognition, *negative FER* mean recognition score for anger, fear and sadness, *SDQ* strengths and difficulties questionnaire

### Emotion recognition

Emotion recognition ability at pre-test was negatively correlated with total SDQ score, *r*_s_(60) = − 0.321, *p* = 0.011, at pre-test. At pre-test, participants in the BP+ and BP− groups differed significantly in the recognition of negative emotions, Welch’s *F*(1, 60) = 55.788, *p* < 0.001, $\eta_{{\text{p}}}^{2}$ = 0.353, but not in the recognition of neutral, *F*(1,60) = 2.032, *p* = 0.159, $\eta_{{\text{p}}}^{2}$ = 0.033, or happy expressions, *F*(1,60) = 0.401, *p* = 0.529, $\eta_{{\text{p}}}^{2}$ = 0.007.

**Fig. 2:**
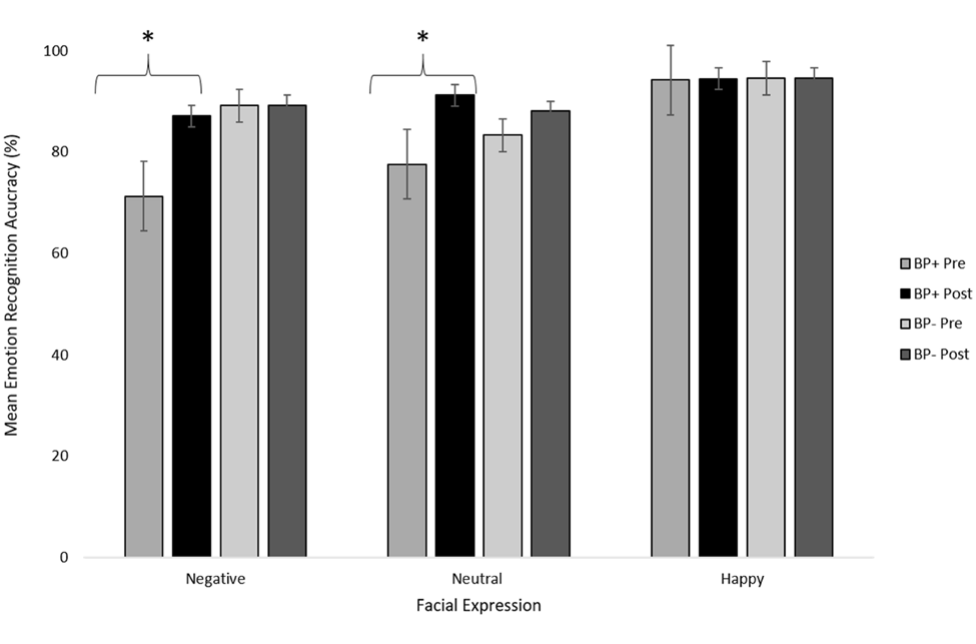
Mean recognition scores for negative, neutral and happy expressions at pre- and post-test. Error bars represent ± 1 standard error. *p < 0.01

Comparing pre-test and post-test scores**,** participants in the BP+ group showed a significant improvement in the recognition of both negative, *t*(39) = − 6.581, *p* < 0.001, *d* = 1.041, 95% CI [− 21.42, − 11.34], and neutral expressions, *t*(39) = − 3.230, *p* = 0.003, *d* = 0.511, 95% CI [− 5.14, 3.47]. Considering the negative emotions individually, the BP+ group showed significant improvements in the recognition of fear, *t*(39) = − 2.120, *p* < 0.001, *d* = 0.340, 95% CI [− 29.79, − 14.23], sadness, *t*(39) = − 5.729, *p* = 0.041, *d* = 0.887, 95% CI [− 16.71, − 0.38], and anger, *t*(39) = − 5.350, *p* < 0.001, *d* = 0.857, 95% CI [− 23.56, − 10.63]. There was no difference in the ability to recognise happy expressions, *t*(39) = − 0.392, *p* = 0.697, *d* = 0.062, 95% CI [− 5.14, 3.47]. In contrast, participants in the BP- group showed no significant difference in their ability to recognise negative, *t*(21) = 0.772, *p* = 0.449, *d* = 0.164, 95% CI [− 2.57, 5.61], neutral, *t*(21) = − 2.049, *p* = 0.053, *d* = 0.436, 95% CI [− 8.39, 0.06], or happy expressions, *t*(21) = 0.420, *p* = 0.678, *d* = 0.090, 95% CI [− 4.48, 6.76] (see Fig. 2). Considering the negative emotions individually, there was no significant improvement in recognising fear, t(21) = − 0.569, *p* = 0.575, d = 0.034, 95% CI [− 8.49, 9.94], sadness, t(21) = 0.163, *p* = 0.872, *d* = 0.119, 95% CI [− 6.73, 3.84], or anger, t(21) = 0.107, *p* = 0.916, *d* = 0.022, 95% CI [− 6.64, 7.36].

### Links between emotion training and change in behavioural problems and well-being

Participants in the BP+ group had significantly lower SDQ scores, *t*(39) = 2.866, *p* = 0.007, *d* = 0.45, 95% CI [1.00, 5.82] at 6-month follow-up, compared to pre-test. By contrast, the change in SDQ scores of the participants in the BP- group was not significant, *t*(21) = 1.448, *p* = 0.162, *d* = 0.31, 95% CI [− 1.03, 5.76] (see Table 2). Furthermore, in the BP + group there was a significant reduction in the percentage of children labelled as ‘at high risk’ for future mental health problems, from 93% at pre-test to 64% at 6-month post-test, *X*^2^(1) = 7.692, *p* = 0.006.

**Table 2:** Mean total SDQ score, with standard deviations in parentheses, at pre- and 6-month post-test for BP+ and BP− participants

	Pre-test total SDQ score	Post-test total SDQ score	*p*
BP+	18.79 (5.16)	15.38 (6.29)	0.007
BP−	15.05 (6.03)	12.68 (6.93)	0.162

### Were changes in emotion recognition associated with changes in problematic behaviour and mental health?

Taking the sample as a whole, there was a significant positive correlation between change in emotion recognition ability and change in SDQ score, *r*(60) = 0.366, *p* = 0.003, indicating that extent of improvement in emotion recognition was associated with the extent of reduction in reported difficulties.

The extent to which SDQ scores at 6-month follow-up were predicted by improvements in emotion recognition was analysed by hierarchical regression analysis involving the full sample of 62 children. At step 1, we regressed 6-month follow-up SDQ scores on pre-test SDQ scores, pre-test FER scores, and IQ. Although the resulting regression equation did not exceed conventional significance thresholds, *F*(3,58) = 2.59, *p* = 0.061, there was one significant predictor, namely pre-test SDQ, *β* = 0.270, *p* = 0.038. At step 2, we entered post-test FER scores as a predictor. Now the overall regression equation was significant, *F*(4,57) = 3.51, *p* = 0.013, and the increase in explained variance (from 11.8% to 19.7%) was also significant, *F*(1,57) = 5.63, *p* = 0.021, *f*^2^ = 0.10. In addition to pre-test SDQ score, *β* = 0.313, *p* = 0.014, post-test FER score was a significant predictor, *β* = − 0.305, *p* = 0.021. Thus, post-test FER score added significantly to the prediction of 6-month SDQ score, after controlling for pre-test SDQ score, pre-test FER score and IQ. A similar pattern of results was found when an identical regression analysis was conducted only on the BP + sample of 40 children: once again the addition of post-test FER score added significantly, *β* = − 0.385, *p* = 0.031, to the explanation of variance in the 6-month follow-up SDQ score (from 5.4% to 17.3%, *f*^2^ = 0.14), after controlling for pre-test SDQ score, pre-test FER score, and IQ.

## Discussion

We examined the role of emotion recognition impairments in the severity of behavioural problems in children with adverse childhood experiences both before and 6 months after receiving interventions, including one that specifically targeted emotion recognition problems. The results replicate previous research showing a relationship between severity of emotion recognition impairments and severity of disruptive behaviour. The results also indicate that improved emotion recognition was associated with longer-term improvements in behaviour and well-being. Impairments in emotion recognition have been implicated in a range of psychiatric disorders [6], and a specific impairment in negative emotion recognition has been widely reported in antisocial and violent samples [22]. Emotion recognition is essential for social functioning and the development of interpersonal relationships [18]. There is therefore good reason to believe that improving emotion recognition in those who display behavioural problems would reduce problematic behaviour.

The extent of improvement in emotion recognition was significantly related to the extent of improvement in SDQ scores, and the results of hierarchical regression analyses showed that emotion recognition measured after the emotion recognition training added significantly to the explanation of SDQ scores at the 6-month follow-up, after taking account of individual differences in pre-test SDQ score, pre-test emotion recognition and IQ. The differences over time were not only statistically significant; they were also of medium effect size. This applies to the change in SDQ scores observed in the training group (*d* = 0.45) and to the additional variance explained by adding post-test FER score to the prediction of 6-month follow-up SDQ scores (*f*^2^ = 0.10). It therefore seems reasonable to conclude that the significant improvement in emotion recognition arising from the emotion training intervention was associated with the reduction in problematic behaviour 6 months after the emotion training.

The Cardiff Emotion Recognition Training (CERT) was delivered in a targeted manner, i.e., to those children exhibiting behavioural problems and impaired emotion recognition (BP+). Their changes in emotion recognition ability following the CERT and in behaviour and well-being 6 months later were assessed both in this group and in a group of children who exhibited behavioural problems but without impaired emotion recognition (BP−), who also continued to receive support from the Early Intervention Partnership Hub. Although the present study lacked sufficient power to detect an interaction between group membership and change in SDQ score (power analysis showed that four times as many participants to test this would have been needed; [9], it is worth noting that only the children who received the CERT showed a significant increase in emotion recognition ability and—more importantly—a significant decrease in SDQ scores as reported by teachers who were unaware that these children had received the CERT. The percentage of children in the BP+ group who were classed as being at high risk for future mental health problems reduced from 93 to 64%.

The present study adds to previous evidence that emotion recognition training is associated with an improvement in longer term criminal behaviour in juvenile offenders [20] by showing that emotion recognition training can be used as an early intervention in younger children exhibiting problem behaviour and that the extent of their emotion recognition improvement is linked to their longer-term mental health and well-being. Whereas previous intervention research by Penton-Voak et al. [33] only targeted the modification of hostile biases, the present study successfully targeted impaired emotion recognition more generally. The fact that recent studies have found no evidence of a hostility bias in disruptive children [41], adolescents with CD [1], or violent offenders [28], whereas impairments in negative emotion recognition were observed in all these studies, indicates that a more general emotion recognition intervention is needed.

Dadds et al. [8] found that the effectiveness of their emotion recognition training in improving behaviour was limited to children with CU traits; however, this effect was not confirmed by independent reports, and the fact that emotion recognition was not shown to improve makes it unclear what was responsible for the reported change in behaviour. By contrast, in the present study children who received the CERT exhibited an improvement in both emotion recognition and longer-term behaviour. The improvement in behaviour was assessed by ratings made 6 months after the training by teachers who were unaware which children had received the CERT. Furthermore, behaviour at the 6-month follow-up was significantly predicted by post-test emotion recognition ability. This suggests that it is possible not only to target a key process linked to mental health and well-being, but also to alter developmental trajectories in antisocial behaviour.

Many existing interventions aiming to reduce behaviour problems in young children focus on improving the child-parent relationship. The effectiveness of certain parenting programs has been demonstrated and they are recommended by the National Institute for Health and Excellence (NICE [37]). However, some parents cannot or will not participate in these interventions [29], and this was also the case in the current sample. The interim evaluation of the early intervention programme in which these children were taking part [10] found that many parents were unable to engage with services, due to mental health issues or substance misuse. Schools provide an ideal setting to deliver interventions, especially to children from adverse backgrounds [19]. At a time when funding services for children and young people are being cut [4]—with an estimated 1.6 million English children being “invisible” to social support [5]—easily delivered, computerised interventions like the CERT are key to increasing the reach of treatments and preventing the escalation of problems in at-risk groups.

We should acknowledge limitations in the present study. Although all children referred into the intervention programme were at high risk for future antisocial behaviour, once the children had been assessed for emotion recognition problems and assigned to their training condition, the two groups were found to differ in severity of behavioural problems. Although this poses analytic challenges, it is consistent with the view that emotion recognition plays a role in severity of behavioural problems. A further limitation is that participants were not randomly allocated to one of the training conditions. Our objective was to deliver the training according to objectively assessed need [26]. Offering emotion recognition training to children who did not have impaired emotion recognition would not only have been a waste of scarce resources but would also have conflicted with the principles of the intervention programme, which state that children should receive the interventions that they need.

The fact that the two groups differed in problem behaviour at the outset raises the possibility that the significant improvement in the emotion training group at follow-up simply reflects regression to the mean [2]. However, we believe that this is unlikely to have been the case here. First, examination of a scatterplot in which change in SDQ scores was plotted against pre-test emotion recognition scores showed no evidence that those with low pre-test emotion recognition scores were especially likely to show behavioural improvement (see Supplementary Figure). Secondly, not only was the extent of the improvement in behaviour significantly associated with the extent of improvement in emotion recognition, but hierarchical regression analyses also showed that post-test emotion recognition scores significantly added to the prediction of SDQ scores at the 6-month follow-up after controlling for pre-test SDQ score, pre-test emotion recognition score and IQ. These analyses show that the change in emotion recognition ability as a result of the emotion training was significantly associated with the observed behavioural improvements.

Children in the non-training group also showed some evidence of behavioural improvement (albeit non-significant) at the 6-month follow-up. Because this group was smaller, it might be thought that lesser power to observe an effect might was responsible for this improvement not being significant. However, the effect size in this group, which is independent of the influence of sample size [13], was noticeably smaller than that for the emotion training group. Moreover, it is worth noting that both groups continued to receive other interventions that might have been responsible for the improvement in teacher ratings in the group who did not receive the emotion training.

Notwithstanding our comments on regression to the mean and on behavioural improvements in the non-training group, an obvious next step in establishing the effectiveness of the CERT would be to allocate children randomly to training and non-training conditions, preferably using a closely matched control intervention. This would rule out the possible role of third variables and enable researchers to arrive at stronger conclusions about the causal impact of the CERT intervention.

A final limitation to acknowledge is that although there was a significant reduction in the number of children in the BP+ group who were rated as being at high risk for psychopathology 6 months after receiving the CERT, two-thirds of these children were still considered as high risk at this stage, showing that further work with these children is needed.

Researchers have challenged the notion that high-risk children inevitably become adult offenders [32, 38], arguing that well-targeted interventions could create a turning-point in antisocial behaviour for high-risk juveniles. The period between childhood and early adolescence is a time when children are particularly adept at social and emotional learning. This creates a window of opportunity for interventions such as the one we have developed and provides a natural opportunity to promote prosocial development in high-risk children.

## Electronic supplementary material

Below is the link to the electronic supplementary material.

Supplementary file1 (DOCX 16 kb)
