# Supplementary material for: Improving emotion recognition is associated with subsequent mental health and well-being in children with severe behavioural problems
Source: Eur Child Adolesc Psychiatry. 2020 Sep 30;30(11):1769–77. doi: 10.1007/s00787-020-01652-y (PMC8558267; doi:10.1007/s00787-020-01652-y)
Supplement: Supplementary file 1 — Supplementary file1 (DOCX 16 kb) [file 787_2020_1652_MOESM1_ESM.docx]

**Supplementary materials:**

**Figure 1: Change in SDQ plotted against pre-test FER score for negative and neutral emotion recognition**

Note: Four BP+ datapoints are overlapping.
